# Diffusion Tensor Imaging and Decision Making in Cocaine Dependence

**DOI:** 10.1371/journal.pone.0011591

**Published:** 2010-07-16

**Authors:** Scott D. Lane, Joel L. Steinberg, Liangsuo Ma, Khader M. Hasan, Larry A. Kramer, Edward A. Zuniga, Ponnada A. Narayana, Frederick Gerard Moeller

**Affiliations:** 1 Department of Psychiatry and Behavioral Sciences, University of Texas Health Science Center at Houston, Houston, Texas, United States of America; 2 Program in Neuroscience, Graduate School of Biomedical Sciences, University of Texas Health Science Center at Houston, Houston, Texas, United States of America; 3 Department of Diagnostic and Interventional Imaging, University of Texas Health Science Center at Houston, Houston, Texas, United States of America; Chiba University Center for Forensic Mental Health, Japan

## Abstract

**Background:**

Chronic stimulant abuse is associated with both impairment in decision making and structural abnormalities in brain gray and white matter. Recent data suggest these structural abnormalities may be related to functional impairment in important behavioral processes.

**Methodology/Principal Findings:**

In 15 cocaine-dependent and 18 control subjects, we examined relationships between decision-making performance on the Iowa Gambling Task (IGT) and white matter integrity as measured by diffusion tensor imaging (DTI). Whole brain voxelwise analyses showed that, relative to controls, the cocaine group had lower fractional anisotropy (FA) and higher mean of the second and third eigenvalues (λ⊥) in frontal and parietal white matter regions and the corpus callosum. Cocaine subjects showed worse performance on the IGT, notably over the last 40 trials. Importantly, FA and λ⊥ values in these regions showed a significant relationship with IGT performance on the last 40 trials.

**Conclusions:**

Compromised white matter integrity in cocaine dependence may be related to functional impairments in decision making.

## Introduction

Decision-making processes are notably faulty in individuals with substance use disorders (SUD). Patterns of decision making in SUD are characterized by preference for alternatives featuring high-risk, high-reward, short-term gains—despite these options being less adaptive with regard to overall expected value [1], [2]. Individuals with SUD appear hypersensitive to reward, undersensitive to loss, and deficient in integrating information across repeated outcomes [2], [3], [4], [5].

The neural correlates that subserve decision making may involve reward valuation in amygdala and ventral striatum [6], [7], [8], [9], and outcome and emotional processing in orbital/prefrontal cortex [10], [11], anterior cingulate cortex [12], [13], [14], and insula [15], [16]. Collectively, these regions are believed to form a neural circuit that functions to facilitate putative stages in the decision-making process, including evaluation of available alternatives, selection and performance of an action, and processing of the outcome [10], [13], [17], [18].

While fMRI studies describing the neural correlates of decision making in substance abusers have been considerable, less is known about white matter integrity in SUD and relationship to functional impairments in decision making. This fact reflects, in part, a relative lack of studies on structure-function relationships using diffusion tensor imaging (DTI). Importantly, structural and volumetric deficits in white matter have been revealed in substance dependent populations compared to controls using several brain imaging techniques.

In alcohol-dependent subjects, researchers found reduced fractional anisotropy (FA) in the splenium of the corpus callosum [19]. These findings in FA correlated with working memory performance. Similar white matter compromise has been observed in cocaine dependence, including decreased white matter volume in prefrontal cortex [20]; diminished age-related increase in frontal lobe white matter [21]; and diminished white matter integrity in the inferior frontal lobes [22], [23]. Moeller et al. [24] reported reduced FA in genu and rostral body of the corpus callosum of cocaine users relative to control subjects. Importantly, reduced FA values were correlated with functional performance deficits on a response inhibition task measuring impulsivity. Moeller et al. [25] found reduced FA in corpus callosum (CC) subregions in cocaine-dependent subjects and increased radial diffusivity—a putative marker for diffusion of water molecules across the axonal membrane, and suggestive of alteration of white matter myelin [26], [27], [28], although the interpretation of “radial diffusivity” may present significant difficulties under certain conditions [29]. More recently, we reported higher mean diffusivity in the posterior CC (isthmus), increased radial diffusivity in the rostral body, and lower FA in the splenium of cocaine-dependent subjects relative to controls [30]. Importantly, our previous work [24], [25], [30] examined mean values of DTI measures within geometrically-defined CC subregions.

The present study examined white-matter integrity in SUD using a different analysis technique: voxelwise analysis using Tract Based Spatial Statistics (TBSS) [31]. Some advantages of voxelwise analysis are that it allows one to find changes anywhere in the brain white matter without needing to pre-specify regions that may be somewhat arbitrarily defined. TBSS was used for voxelwise analysis because TBSS may overcome potential problems with the registration and alignment of white matter between subjects in order to allow cross-subject statistical analysis [31]. TBSS attempts to achieve this by projecting each subject's FA data onto an FA skeleton that represents the white-matter fiber bundles that are common to all the subjects in the study [31].

Corresponding to known fMRI and DTI abnormalities, cocaine and methamphetamine abusers also have shown marked deficits in decision-making processes [1], [2], [32], [33]. While these decision making-impairments have been studied intensively under fMRI protocols, they have not been investigated with regard to abnormalities in white matter integrity. Given the role of white matter fiber tracts in facilitating the connection of neural circuits and regions, we hypothesized that, in cocaine-dependent vs. controls subjects, compromises in white matter integrity as revealed by the TBSS analysis would be associated with functional impairment in decision making as measured by the Iowa Gambling Task [2].

## Methods

### 2.1 Subjects

This study was approved by the Committee for the Protection of Human Subjects of the University of Texas Health Science Center at Houston, and was performed in accordance with the Declaration of Helsinki. The consent process and all procedures were reviewed and approved by the institutional review board (IRB) at the University of Texas Health Science Center at Houston prior to initiating studies. Written informed consent was obtained from all subjects by investigators and research staff.

Subjects with current cocaine dependence and non-drug using normal control subjects were recruited through advertisements for research volunteers. All subjects were screened for psychiatric and nonpsychiatric medical disorders using the Structured Clinical Interview for DSM-IV [34], a medical history, physical examination, blood chemistry, and complete blood count. On all test days, and immediately prior to the MRI scan, breath alcohol (Alcosensor III, Intoximeters, Inc), urine drug screening (UDS), and pregnancy testing were conducted. UDS was carried out using enzyme multiple immunoassay (EMIT d.a.u ® -SYVA), which tested for cocaine, THC, amphetamine, methamphetamine, and opiates (Syva Inc). The Addiction Severity Index [35] was used to document lifetime drug and alcohol use. Subjects were excluded for the following: current or past Diagnostic and Statistical Manual of Mental Disorders Fourth Edition (DSM-IV) [36] Axis I disorders other than substance abuse or dependence; medical disorders that may affect the central nervous system; a positive breath alcohol test; or a positive pregnancy test result (females). Prior to conducting the study analysis, two control subjects and one cocaine-dependent subject were excluded due to clinical abnormalities on Fluid-Attenuated Inversion Recovery (FLAIR) MRI. The cocaine subject who was excluded had a finding of several hyperintensities in the right frontal white matter most likely representing microvascular disease according to the radiologist's report. One of the two control subjects who were excluded had a finding of mild prominence of the subarachnoid space over the cerebral convexities representing cerebral atrophy according the radiologist's report. The other control subject who was excluded had a finding of retention cyst or retained mucous seen in the left lateral sphenoid sinus. Because this abnormality was not in the brain per se, and the subject was asymptomatic, this subject was erroneously excluded due to technical error. All three subjects who were excluded were informed about the findings and referred to their primary-care physicians for follow-up.

A total of 19 cocaine-dependent subjects and 18 controls were initially tested. Four cocaine-dependent subjects were subsequently excluded due to co-morbid alcohol dependence, which is also related to white matter impairment [19], [37]; leaving 15 cocaine-dependent subjects in the final sample. None of the subjects in the final sample had a history of present or past alcohol dependence. In the final sample, four control and five cocaine subjects were included who had FLAIR scans with a few small white matter hyperintensities that were judged to the clinically insignificant by a radiologist (LAK) and the other physician coauthors (FGM and JLS) prior to any experimental analysis. All other subjects had no brain abnormalities on FLAIR scans. In addition, control subjects were excluded if they had a positive urine drug screen or breath alcohol test, and during screening if they met current or lifetime criteria for any DSM-IV Axis I disorder. For subjects who smoked (see Table 1), nicotine intake and abstinence-related complications were controlled by allowing one cigarette to be smoked approximately 45 minutes prior to the start of the scan [38], [39]. Similarly, subjects who smoked were allowed to smoke one cigarette approximately 45 minutes prior to IGT testing.

**Table 1:** Subject demographics.

Group	Age (years)	Education^a^	Gender	Cocaine Use^b^	Lifetime Alcohol Use^c^	Smoke Status^d^	Subst Abuse/Dependence^e^
Cocaine	38.47±2.20	13.13±0.48	10 M, 5 F	16.1±2.07	87.57±26.43	13/15	sedative = 1
					10.1±1.71		opiate = 2
					11.4±0.75		cannabis = 5
							alcohol = 4^f^
							hallucinogens = 1
							PCP = 1
							stimulant = 2^g^
Control	35.2±2.6	15.44±0.53	9 M, 9 F	0	13.93±4.73	4/18	0

All values represent either number of subjects or the mean±SEM.

a Represents years of education completed. Completion of GED scored as 12 years. The groups were significantly different: t (31)  = 3.20, p = 0.003.

b Cocaine use frequency is presented in three formats: (top) number of days used over the past 30 days; (middle) number of years used (lifetime); and (bottom) the Kreek-McHugh-Schluger-Kellogg scale (KMSK, [72]), a rating designed to quantify drug use. The KMSK scale assesses the frequency, amount, and duration of drug use during the individual's period of heaviest consumption.

c Lifetime total alcohol use in kg, estimated from years of use, weekly frequency of drinking, drinks per occasion, and type of alcohol consumed. The groups were trend significantly different: Wilcoxon Two-Sample z = 1.81, p = 0.071.

d Number of subjects who reported current smoking. Intake data did not contain consistent frequency of use levels across subjects. The groups were significantly different, χ^2^ = 13.60, p<0.001.

e Number of subjects meeting DSM-IV criteria for past or present abuse or dependence of a drug other than cocaine.

f Two subjects had present alcohol abuse only. Two other subjects had past alcohol abuse only. None of the subjects had past or present alcohol dependence.

g Stimulant other than cocaine (e.g., amphetamine); one subject had past abuse and dependence, one had past abuse.

All cocaine-dependent subjects were referred for treatment of cocaine dependence at the end of the study. Cocaine-dependent subjects had a DSM-IV diagnosis of both current and past cocaine dependence. Other substance use disorders (SUD) among the cocaine group are listed in Table 1. None of the control subjects had DSM-IV diagnoses of past or current substance abuse or dependence. Nine of the cocaine subjects had one SUD diagnosis; one cocaine subject had two diagnoses, and nine had more than two diagnoses due to overlap. The present study used a different group of subjects than our previous reports [24], [25].

### 2.2 Decision Making Methodology and Data Analysis

The Iowa Gambling Task, IGT [2] was used to measure decision making. The task is widely used in neurocognitive research [2], [40], [41]. On each of 100 trials, subjects are presented with four physically identical decks of cards shown on a computer screen, labeled A, B, C and D. Subjects are also given a hypothetical $2000 loan to begin the task. Cumulative totals of money borrowed and money gained are shown in text and represented by colored horizontal bars near the top of the screen. Gains and losses on individual trials are shown in text just below the horizontal bars.

Subjects are instructed that on each trail they may choose cards from any one of the four decks. They are not provided information about session length or number of trials per session. They are instructed that each deck produces (hypothetical) money, but some decks also result in a loss of money. The goal of the game is to win as much money as possible and to avoid losing money. Subjects are also told that they cannot determine which specific decks and trials result in money loss, but that some decks are less favorable and should be avoided.

The gains and losses on the decks are fixed in a predetermined arrangement. Decks A and B produce a $100 gain; decks C and D produce a $50 gain. Each deck also results in variable losses: deck A = −$75 to −$150 on 50% of trials; deck B = −$1250 on 10% of trials; deck C = −$25 to −$75 on 50% of trials; and deck D = −$250 on 10% of trials. The A and B (disadvantageous) decks produce greater gains but corresponding greater losses, and a net loss of $250 per 10 trials. In contrast C and D (advantageous) decks produce smaller gains, but also smaller monetary losses, and result in a net gain of $250 per 10 trials. However, the prevalence of the larger losses is loaded toward trials in the latter trials of the session, inviting a preference for (disadvantageous) decks A/B in early trials. Optimal performance on the task requires integrating information over trial-by-trial outcomes, discriminating the advantage of decks C/D over decks A/B, and shifting choice preference to the C and D decks. An abundance of data has demonstrated that healthy controls typically sample cards from each deck over the first 20 trials, then, between trials 20 and 40, begin to more consistently choose the advantageous decks. Patients with orbitofrontal cortex damage and substance use disorders generally continue to favor the disadvantageous decks [2], [41], [42].

On the IGT, two options (advantageous decks) return a positive net gain, and two (disadvantageous decks) return a net loss. Data analyses examined quality of decision making as measured by the net score (choice of advantageous decks - disadvantageous decks) across five 20-trial blocks, and the overall net score (across 100 trials). IGT net score data were analyzed via mixed-model ANOVA. The model included one between-subjects factor (two groups) and one repeated measures factor (five 20 trial blocks). Overall net score was assessed via t-test. The relationships of DTI measures with decision-making performance were analyzed with mixed-model ANOVA (SAS 9.2 Mixed Procedure) and linear regression analysis (SAS 9.2 Regression Procedure).

In addition to the analyses described above, a computational analysis of decision-making patterns was conducted in order to characterize the trial-by-trial decision making patterns on the IGT. The expectancy valence model (EVM) was employed. The EVM is a mathematical model of decision-making processes, as detailed in [4], [5], [43], [44], [45]. It includes three parameters, which represent putatively independent aspects of the decision-making process (uncorrelated in the present dataset). These parameters are labeled *w, a*, and *c*. The *w* parameter represents the weight, or relative valence, given to the consequences (gains and losses) of each decision. The parameter *a* represents the rate at which the valence (*w*) is updated, and corresponds to the influence of recent vs. more distant outcomes of each decision. The consistency or *c* parameter determines the sensitivity of the decision maker's choices, determined by the consistency with which the expectancy value (determined by *a* and *w*) is applied to each decision. Model parameters are adjusted following the outcome of each trial as value of the options are recalibrated, corresponding to the way a human decision maker may have an affective reaction to gain or loss. Parameter values were compared across groups using ANOVA models as described above with the IGT net score.

### 2.3 Brain Imaging Methodology and Data Analysis

Whole brain DTI data were acquired on a Philips 3.0 T Intera system with a six-channel receive head coil (Philips Medical Systems, Best, Netherlands). DTI images were acquired in the transverse plane using a single shot spin-echo diffusion sensitized echo-planar imaging (EPI) sequence (21 gradient directions plus B_0_ image, b-factor = 1000 s/mm^2^, repetition time = 6100 ms, echo time = 84 ms, 44 contiguous axial slices, field-of-view = 240 mm×240 mm, 112×112 acquisition matrix, 256×256 reconstructed matrix, 0.9375 mm×0.9375 mm reconstructed in-plane resolution, slice thickness = 3 mm. The diffusion tensor encoding scheme is based on the uniformly distributed and balanced rotationally invariant *Icosa21* tensor-encoding set [46]. A SENSE acceleration factor or k-space undersampling R = 2 was used to help reduce EPI image distortions. The diffusion-encoded volumes were acquired with fat suppression. The DTI acquisition time was approximately 7 min and resulted in signal-to-noise ratio independent DTI-measure estimation. Whole brain FLAIR images were also acquired to screen for clinical brain abnormalities.

The DTI images were processed using the FMRIB Software Library: FSL, www.fmrib.ox.ac.uk/fsl, version 4.04 [47], [48]. The DTI images were corrected for eddy current distortions and head motion [49]. Next, the FMRIB's Diffusion Toolbox (FDT/FSL) [50] was used to fit a diffusion tensor model to the data at each voxel. Voxelwise values of the fractional anisotropy (FA), the first eigenvalue (λ_1_), the second eigenvalue (λ_2_), and the third eigenvalue (λ_3_) were derived from the tensor model. The mean of the second and third eigenvalues was calculated as λ⊥ =  (λ_2_+λ_3_)/2. FA measures deviation from isotropy, and may reflect the degree of alignment of cellular structures within fiber tracts, as well as their structural integrity [51]; however this interpretation may be invalid in voxels containing crossing fibers [52]. We also computed λ_1_ and λ⊥ because there is considerable experimental evidence that these two measures improve pathological specificity compared to FA [53]. Although λ_1_ is sometimes referred to as “axial diffusivity” and λ⊥ is sometimes referred to as “radial diffusivity”, the eigenvalues of the diffusion tensor do not always reflect the underlying directionality of the tissue in pathological conditions or in areas of low anisotrophy, partial volume effects, crossing fibers, or other misalignment of the principle eigenvector [29]. Higher λ⊥ may be indicative of greater diffusion perpendicular to the fiber tracts and has been reported to be associated with impairment in myelin [26], [27], [28], [54]; however this association may not always be valid, as indicated above [29]. Lower λ_1_ may indicate less diffusion of water in the direction parallel to the fiber tract and may be associated with axonal damage [28], but this association also may not always be valid, and lower λ_1_ may not necessarily indicate white matter impairment [29].

Whole brain voxelwise statistical analyses of the DTI data (FA, λ_1_, λ⊥) were carried out using Tract-Based Spatial Statistics (TBSS/FSL) [31]. FA data were aligned into the FMRIB58_FA image in Montreal Neurological Institute-152 (MNI152) standard space [31], [55], [56] using the nonlinear registration IRTK [57]. The mean FA image was created and thinned to create a mean FA skeleton representing the centers of all tracts common to all subjects. The FA threshold was set to be FA = 0.20. Each subject's aligned FA data was then projected onto this skeleton. TBSS was also applied to other diffusion tensor-derived measures (λ_1_, λ⊥).

For the TBSS analysis the group differences among the DTI measures were tested voxelwise throughout the whole-brain white-matter skeleton using the FSL nonparametric program, Randomise, with a cluster-forming threshold of t = 2.00, variance smoothing of 5 mm Half Width at Half Maximum, and 10000 random permutations. For all TBSS analyses reported herein, significance level was set as two-tailed family-wise-error (FWE) corrected probability level <0.05 at the cluster level of inference. Based on this significance criterion, the empirically determined cluster size for each significant cluster is reported in Table 2. Within the clusters that showed two-tailed family-wise error-corrected significant differences between groups, we calculated the mean value of the DTI measure across all voxels within the cluster for each subject. These mean values were used to test the correlations between DTI measures and the behavioral data.

**Table 2:** Outcomes of voxelwise whole brain TBSS between-groups analysis (nonparametric t-test) of FA and λ⊥.

Cluster label (color key)	Comparison	# voxels in cluster	FEW p-value	X	Y	Z	Maximal voxel t
FA cluster 1 (Fig. 2, green)	Cocaine<Controls	1808	0.004	34	−44	11	4.635
FA cluster 2 (Fig. 2, blue)	Cocaine<Controls	1162	0.013	−2	10	22	4.127
λ⊥ cluster 1 (Fig. 3, green)	Cocaine>Controls	1998	0.003	24	−29	32	5.396
λ⊥ cluster 2 (Fig. 3, red)	Cocaine>Controls	1035	0.014	−31	−45	15	5.932
λ⊥ cluster 3 (Fig. 3, blue)	Cocaine>Controls	789	0.027	3	17	19	4.066
λ⊥ cluster 4 (Fig. 3, black)	Cocaine>Controls	710	0.034	−25	−33	33	7.092

For each significant cluster, the number of voxels in the cluster, the two-tailed cluster p value, the X, Y, and Z MNI standard space coordinates (mm), and the t value of the relative maximal voxel are provided. Cluster p values reflect the two-tailed family-wise error-corrected (FWE) probability at the cluster level of inference (column labeled FWE p-value). X, Y, and Z values are MNI standard space coordinates (mm). The color key refers to the color coding of each cluster in Figure 2 and Figure 3.

To determine the brain regions that corresponded to significant FA and λ⊥ clusters, we consulted the MRI Atlas of Human White Matter [58], the JHU ICBM DTI-81 and JHU White Matter Tractography atlases [58], [59], [60], and the Montreal Neurological Institute (MNI) structural atlas [55], [56].

## Results

Table 1 provides demographics for the cocaine-dependent and control groups. A total of 15 cocaine dependent subjects and 18 controls were included in the final sample. There were no significant group differences in age: t (35)  = 1.31; gender: χ^2^ (1)  = 2.78; or handedness χ^2^ (1)  = 0.07.

### 3.1 Decision Making Data

**Figure 1:**
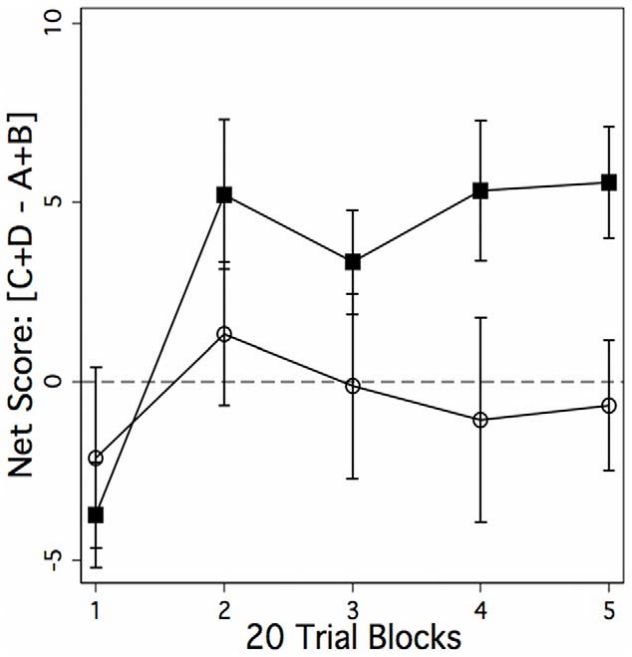
Decision making performance on the Iowa Gambling Task (IGT) for the cocaine- dependent (open circle) and control (black square) subjects. Described in detail in the experimental procedures, each trial on the IGT presents subjects with four choices represented as decks of cards. Two sets (decks C, D) return a positive net gain, and two (A, B) return a net loss. The figure shows the net score (choice of advantageous decks - disadvantageous decks) across 100 test trials, divided into five 20-trial blocks. Beginning between trial 20 and 40, control subjects shifted preference toward decks C and D (net gain), while cocaine dependent subjects continued to divide choices evenly between advantageous and disadvantageous decks, slightly favoring decks A and B (net loss). This between-group difference was statistically significant: F (1, 31)  = 4.32, p = .046.

Mixed model ANOVA revealed a significant between-group difference across blocks: F(1, 31)  = 4.32, p = .046, and a significant within-subject effect of block: F(4, 124)  = 3.69, p = .007. The block x group interaction was not significant: F(4, 124)  = 1.61. Post-hoc tests showed that the groups were significantly different in blocks 4 and 5 (p<.05). Block-by-block means for each group are presented in the supplemental materials (Table S1). Shown in Figure 1, beginning at approximately the 20th trial, control subjects shifted preference toward advantageous decks, whereas cocaine-dependent subjects showed little change in preference over the 100 trials, favoring the disadvantageous decks. A t-test showed that overall net score was significantly different between the groups: t (33)  = 2.05, p = .024.

Results from the computational EVM analyses were in the anticipated direction. Cocaine subjects had higher valence (*w*) scores (.39 vs. .30), higher updating (*a*) rates (.48 vs. .25), and lower consistency (*c*) scores (.12 vs. .35). However, considerable variability in the data rendered all results not statistically significant.

### 3.2 DTI Data: Whole Brain Voxelwise TBSS Analysis

**Figure 2:**
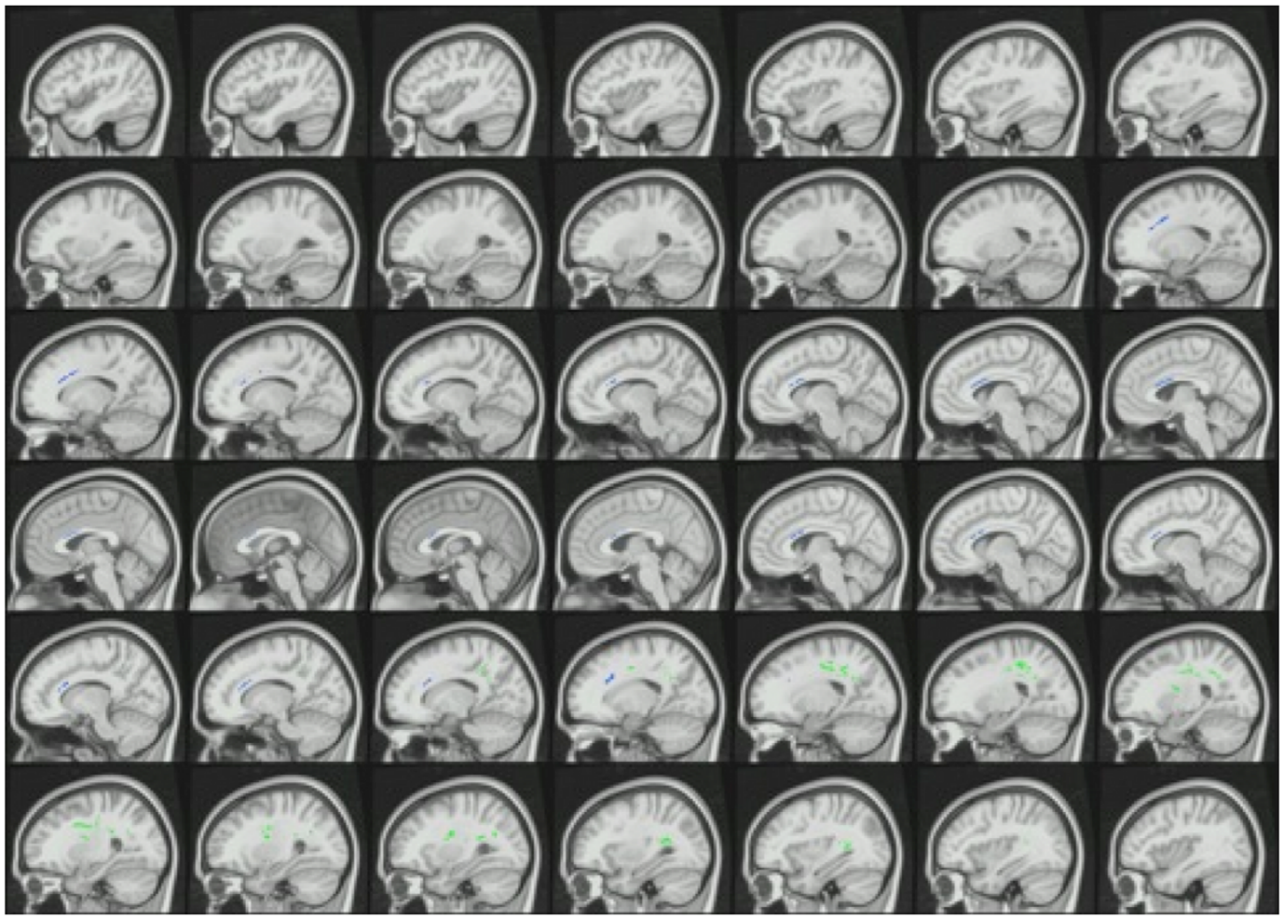
Clusters that had significantly lower fractional anisotropy (FA) in cocaine-dependent compared to control subjects are overlaid in color on a montage of sagittal slices of the MNI152 standard space template T1 brain image. Green voxels represent FA cluster 1 and blue voxels represent FA cluster 2 in Table 2. The slice in the upper left corner is in the left hemisphere; the lower right corner slice is in the right hemisphere. Note that the cluster colors were arbitrarily chosen to identify different clusters and do not represent a scale of t values.

Shown in Table 2, two significant clusters were found in which fractional anisotropy (FA) was significantly lower for cocaine-dependent subjects (Cocaine) than control subjects (Controls). In the first FA cluster, the values were: Cocaine mean FA across all voxels in the cluster = .454±.005 standard error; Controls .506±.006; family-wise error corrected cluster *p* = .004. In the second FA cluster, the values were: Cocaine .557±.010; Controls .613±.009; corrected cluster *p* = .013. Voxels from the first FA cluster (green color in Figure 2) were found mainly in right corticospinal tract and right superior corona radiata in frontal and parietal lobes. Voxels from the second FA cluster (blue color in Figure 2) were found mainly in anterior body of the corpus callosum and bilateral anterior corona radiata in frontal lobe. No significant clusters were found in which Cocaine had higher FA than control subjects.

**Figure 3:**
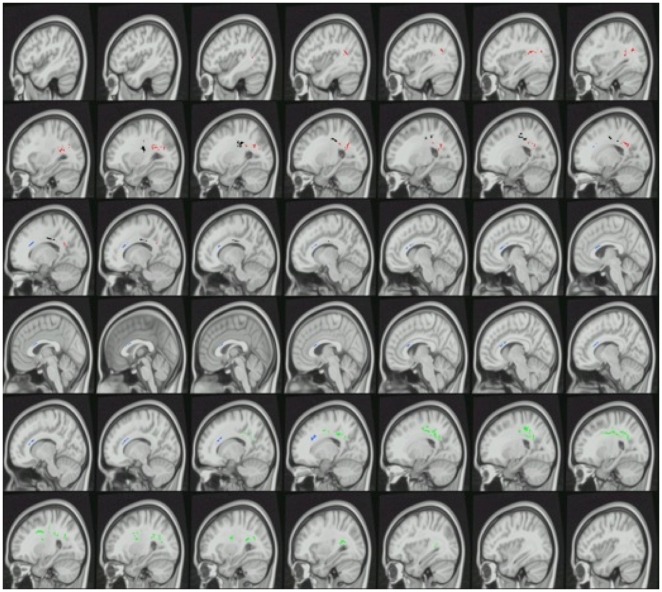
Clusters that had significantly higher mean of the second and third eigenvalues (λ**⊥**) in cocaine-dependent subjects compared to control subjects are overlaid in color on a montage of sagittal slices of the MNI152 standard space template T1 brain image. Green voxels represent λ⊥ cluster 1, red voxels represent λ⊥ cluster 2, blue voxels represent λ⊥ cluster 3, and black voxels represent λ⊥ cluster 4 in Table 2. The slice in the upper left corner is in the left hemisphere; the lower right corner slice is in the right hemisphere. Note that the cluster colors were arbitrarily chosen to identify different clusters and do not represent a scale of t values.

Table 2 also reveals four significant clusters in which mean of the second and third eigenvalues (λ⊥) was significantly greater for cocaine-dependent subjects than controls. In the first λ⊥ cluster, the values were: Cocaine mean λ⊥ across all voxels in the cluster = 571.9 (10^−6^ mm^2^/s) ±7.3 standard error; Controls 519.0±6.4; corrected cluster *p* = .003. In the second λ⊥ cluster, the values were: Cocaine 614.2±8.2; Controls 548.4±9.4; corrected cluster *p* = .014). In the third λ⊥ cluster, the values were: Cocaine 537.4±14.1; Controls 471.3±9.9; corrected cluster *p* = .027. In the fourth λ⊥ cluster, the values were: Cocaine 546.3±7.3; Controls 497.2±5.3; corrected cluster *p* = .034. Voxels from the first λ⊥ cluster (green color in Figure 3) were found in frontal and parietal lobes, mainly in right corticospinal tract and right superior corona radiata. Voxels from the second λ⊥ cluster (red color in Figure 3) were found in parietal lobe and occipital lobe, mainly in left posterior corona radiata, left posterior body of the corpus callosum, left optic radiation, left superior longitudinal fasciculus, left posterior thalamic radiation, and left retrolenticular part of internal capsule. Voxels from the third λ⊥ cluster (blue color in Figure 3) were found mainly in bilateral anterior body of the corpus callosum. Voxels from the fourth λ⊥ cluster (black color in Figure 3) were found mainly in frontal and parietal lobes in left corticospinal tract, left superior corona radiata, left posterior body of the corpus callosum, and left posterior corona radiata. 1351 voxels from λ⊥ cluster 1 (green in Figure 3) overlapped (i.e., were found in common) with FA cluster 1 (green in Figure 2). 713 voxels from λ⊥ cluster 3 (blue in Figure 3) overlapped with FA cluster 2 (blue in Figure 2). There were no other overlaps between λ⊥ and FA clusters. No significant clusters were found in which cocaine-dependent subjects had lower λ⊥ than control subjects. No significant clusters were found in which cocaine-dependent subjects had significantly different first eigenvalue (λ_1_) compared to control subjects.

### 3.3 Relationships between DTI and Decision Making Data

Relationships were examined among IGT net score on blocks 4 and 5 (summed) and all six significant DTI clusters (two FA and four λ⊥, see Table 2), collapsed across groups in order to examine global relationships between the neuroimaging and behavioral findings. We focused on blocks 4 and 5 because these blocks showed significant between-group differences.

For the FA clusters in Table 2, i.e, the clusters where FA had been found to be significantly different between groups, regression analysis was conducted to determine the relationships between the FA values and the decision making measure (IGT score in blocks 4 and 5). Preliminary mixed model analysis (SAS 9.2 Mixed Procedure) of interaction effects was conducted, in which decision making was the independent variable, group was the between subjects factor (2 levels: cocaine dependent subjects and controls), and FA cluster was the within-subjects factor (2 levels: FA clusters 1 and 2 from Table 2). This analysis showed that there were no significant interaction effects of decision making x cluster (F[1,30]  = 0.05, *p* = 0.8308), decision making x group (F[1, 30.4]  = 0.20, *p* = 0.6564), or decision making x group x cluster (F[1,30] = 0.17, *p* = 0.6790), indicating respectively that there were no significant differences between the clusters in the regression slopes of FA on decision making, and no significant differences between groups in the regression slopes of FA on decision making across and between the clusters. In the reduced model after removing the group and interaction factors, mixed model analysis of the regression of FA on decision making showed a significant main effect of decision making across the two FA clusters (F =  [1,31]  = 7.01, *p* = 0.0126, R^2^ = 0.184).

Similarly, for the λ⊥ clusters in Table 2, i.e., the clusters where λ⊥ had been found to be significantly different between groups, regression analysis was conducted to determine the relationships between the λ⊥ values and the decision making measure (IGT score in blocks 4 and 5). Preliminary mixed model analysis of interaction effects was conducted in which decision making was the independent variable, group was the between subjects factor, and cluster was the within subjects factor (4 levels: λ⊥ clusters 1, 2, 3, and 4 from Table 2). This analysis showed that there were no significant interaction effects of decision making x cluster (F[3,30]  = 0.27, *p* = 0.8465), decision making x group (F[1,29]  = 0.00, *p* = 0.9858), or decision making x group x cluster (F[3,30]  = 0.27, *p* = 0.8488), indicating respectively that there were no significant differences between the clusters in the regression slopes of λ⊥ on decision making, and no significant differences between groups in the regression slopes of λ⊥ on decision making across and between the clusters. In the reduced model after removing the group and interaction factors, mixed model analysis of the regression of λ⊥ on decision making showed a significant main effect of decision making across the four λ⊥ clusters (F =  [1,31]  = 11.99, *p* = 0.0016, R^2^ = 0.279).

Based on the results of the above analyses, post-hoc regression analysis (SAS 9.2 Regression Procedure) was conducted separately for each individual cluster in Table 2. Outcomes of each regression were corrected for multiple comparisons (e.g., for the six post-hoc cluster analyses) using the false discovery rate (FDR). These analyses showed significant regressions of FA on decision making for FA cluster 2 (F[1,31]  = 7.04, uncorrected *p* = 0.0125, FDR corrected *p* = 0.0250, R^2^ = 0.185); for λ⊥ cluster 1 (F[1,31]  = 9.90, uncorrected *p* = 0.0036, FDR corrected *p* = 0.0216, R^2^ = 0.242); and for λ⊥ cluster 4 (F[1,31]  = 7.57, uncorrected *p* = 0.0098, FDR corrected *p* = 0.0250, R^2^ = 0.196). The remaining three clusters did not show significant individual regressions (uncorrected *p*>0.05).

**Figure 4:**
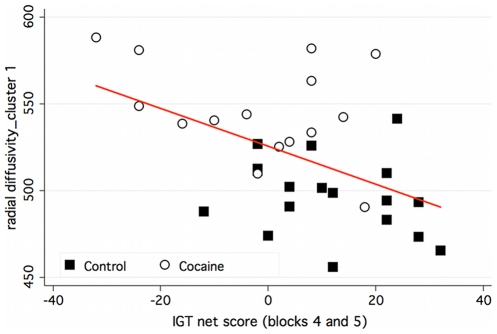
Graphical presentation of the relationship between performance during blocks 4 and 5 (summed) on the IGT and the DTI for λ**⊥** cluster 1 (see Table 2 for X, Y, Z coordinates and Figure 3 for brain image). Cocaine-dependent subjects are represented as white circles; control subjects as black squares. λ⊥ is expressed as 10^−6^ mm^2^/s. The solid red line shows the linear fit to the data for both the cocaine-dependent and control subjects; depicting a relationship in which λ⊥ values decline as a linear function of IGT net score on blocks 4 and 5 (*R^2^* = .24).

The relationship between DTI and decision making is represented graphically in Figure 4, which shows the relationships between the λ⊥ cluster 1 and the summed IGT net score on blocks 4 and 5. In general, the findings indicate that poorer IGT performance (e.g., more choices from disadvantageous decks) was associated with a greater mean of the second and third eigenvalues (suggesting disruption of axonal myelination). This relationship provides evidence consistent with the hypothesis that compromised white matter integrity was related to functional impairment in decision making. By extension, these impairments were exacerbated in individuals with cocaine dependence.

There were a number of variables within and between the cocaine and control groups that could have plausibly influenced the DTI data, thereby biasing the results. These included alcohol use, education completed, smoking status, and (within the cocaine group) age of onset of cocaine use.

Group differences were observed in level of completed education (Table 1). Additionally, correlation analyses were conducted to assess relationships between education level and each of the six significant DTI outcomes (two FA clusters and four λ⊥ clusters). These data are presented in the supplemental materials (Table S2). Four correlations were found with uncorrected *p* less than .05 (two FA and two λ⊥ clusters; no clusters were significant after correction for false discovery rate). Due to these outcomes, the DTI data were re-analyzed using the TBSS General Liner Model (GLM) regression analysis with education level as a factor. To avoid co-linearity (e.g., group and education level were strongly related), the analyses were conducted separately for each group. Within each group, no significant clusters were related to education level (all two-tailed corrected *p*>.05).

Within the cocaine-dependent group, there was a notable range in age of onset of regular cocaine use (range = 19 years 11 months to 41 years 7 months). In the same manner as education, the DTI data were analyzed using TBSS GLM regression analysis with age of onset as a factor. There were no clusters for which there was a significant regression with age of onset of cocaine use and the DTI values (all two-tailed corrected *p*>0.05).

Alcohol use was addressed by initially removing the four comorbid alcohol-dependent subjects from the cocaine-dependent group. In addition, there were no significant Spearman correlations (uncorrected *p*>.05) between lifetime mass of alcohol use and mean FA or λ⊥ within each of the 6 clusters listed in Table 2 for either the cocaine dependent group or the control group. In the same manner as education, the DTI data were analyzed using TBSS GLM regression analysis voxelwise throughout the whole brain with lifetime mass of alcohol use as a factor. There were no clusters for which there was a significant regression with lifetime mass of alcohol use and the DTI values (all two-tailed corrected *p*>.05).

As shown in Table 1, smoking status differed significantly between the groups. To assess the possible influence of smoking status, as well as other potentially significant predictors, multiple linear regression analyses were conducted with the following variables: smoking status, education level, group, and IGT net score. These variables were regressed against each of the individual significant DTI clusters (two FA and four λ⊥, see Table 2); thus six regression models were examined. Across the six regression analyses, only group status was a significant predictor (*p*<.05). Smoking and education were uniformly weak predictors (*p* values>.4). These regression analyses were repeated individually within each group, with group removed as a factor and age of onset added as a factor in the cocaine group. The results were largely consistent with the combined models; none of the factors were significant predictors of the DTI values.

## Discussion

We discovered a relationship between impaired decision making and DTI-measured white matter integrity in frontal and parietal regions lateral to the corpus callosum: i.e., in superior longitudinal fasciculus, corticospinal tract, and superior corona radiata. To our knowledge, this is the first such demonstration in cocaine dependent individuals. The data complement a previous SPECT-based report showing that impaired IGT performance was related to perfusion in frontal gyri and anterior cingulate gyrus [61].

The association between the DTI measure of the mean of the second and third eigenvalues and decision-making deficits on the IGT suggests that putatively compromised white matter may have functional consequences on decision making processes in cocaine abusers, or vice versa. This is consistent with fMRI data in drug dependent vs. healthy control populations that revealed BOLD differences in brain regions implicated in decision making, including limbic structures, insular cortex, and prefrontal and frontal cortex [62]. Notably, both fMRI and focal lesion studies have suggested that insula and prefrontal cortex are critical to performance on the IGT [63], [64], [65].

There has been much concern recently in the neuroimaging literature regarding mistakes of circular analysis or “double-dipping” that can invalidate results [66] and the related problem of over-inflated correlations due to non-independent analysis [67]. Because the present study selected voxels for subsequent regression analyses, the possibility of circular or non-independent analysis should be carefully considered. According to Vul et al. [67], over-inflated correlation due to non-independence occurs when researchers select voxels based on a correlation analysis conducted voxel-by-voxel over the whole brain with the behavioral measure of interest, then report or plot the observed correlation from just those voxels that have a significant correlation. In other words, the voxels are selected for presentation of the correlation coefficient because they correlated highly with the behavioral measure of interest. Vul et al. [67] suggested that researchers use methods for selecting voxels that do not produce non-independent analyses. According to Vul et al. [67], one method to avoid the non-independence error is to select the voxels in a principled way that is blind to the correlations of those voxels with the behavioral measure, for example to select those voxels that are significantly active for a task contrast, and once the set of voxels is selected by this criterion, one number should be obtained across those voxels for each subject, such as the mean signal change, for subsequent correlational analysis [67]. Although the present study did not involve functional activation, the present study followed an approach analogous to that suggested by Vul et al. [67], in which an anatomical contrast (of DTI measures) between groups was used to select the voxels of interest blind to the correlation of those voxels with the behavioral measure, and then the mean DTI values across those voxels for each subject was used in the subsequent regression analyses.

Kriegeskorte et al. [66] warn that selection of voxels on the basis of functional activation differences can lead to circular analysis and bias because if the selection process is based on the design matrix it creates dependencies between the experimental design and noise in the selected data which violates the assumption of random sampling. This is a type of “double dipping,” in which using the same data for selection and selective analysis results in invalid statistical inference if the test statistics are not independent of the selection criteria under the null hypothesis [66]. Kriegeskorte et al. [66] describe methods for achieving non-circular analysis. If it can be demonstrated that all statistical results are independent of the selection process under the null hypothesis, for example, if a region of interest is defined on the basis of brain anatomy, “then we can argue that the selection of functional data by this criterion cannot possibly bias the results statistics” ([66], supplementary discussion). In the present study, the voxels were selected based on anatomical rather than functional differences in DTI measures between groups because the DTI scan did not measure functional activation, and there was no behavioral task or behavioral activation involved with the selection of voxels for subsequent behavioral regression analysis. Thus, there was no known dependency of the selection process with the regression analysis in the present study. Because of the possibility that there may still lurk subtle or hidden dependencies that are at present unknown, the present regression results should be replicated using the current regions of interest with a completely different set of subjects. Nevertheless, aside from the regression outcomes, the differences in DTI measures per se that were found between the cocaine-dependent group and the control group in the present study do not follow as the result of circular or non-independent analysis according to the guidelines given in [66], [67].

Evidence of putatively reduced white matter integrity was found in the rostral body and isthmus of the corpus callosum. These results partially replicate our previous findings [25], [30] and support a growing body of evidence suggesting compromised white matter integrity in the corpus callosum in individuals with cocaine dependence [20], [22], [23], [24]. If white matter is indeed susceptible to insult by repeated cocaine exposure, the corpus callosum may be a candidate marker for measurement of compromised white matter in cocaine abusers, due to the dense volume of white matter fibers in this pathway [58].

Uniquely, we identified putatively compromised white matter in frontal and parietal regions lateral to the corpus callosum; i.e., in superior longitudinal fasciculus, corticospinal tract, and superior corona radiata. The association fibers of the superior longitudinal fasciculus may be connected (directly or indirectly) to relevant cortical regions [58] that subserve decision making (e.g., insula, frontal cortex). The data could therefore be a marker for impairment in decision making following chronic cocaine use. These hypotheses are speculative, however, as (a) relationships between gray and white matter connectivity and function in these regions are at present unclear, (b) these fiber tracts connect large segments of the brain [58] that may be nonspecific with regard to decision making circuits identified using fMRI, and (c) these data do not address whether the DTI findings are related to the sequelae of chronic cocaine use, pre-existing conditions, or the interaction of these factors. Future studies may endeavor to examine structure-function relationships more precisely using fiber tracking and connectivity analyses in integrated structural-functional MRI experiments during ongoing decision making (e.g., [68]).

The finding of higher mean of the second and third eigenvalues in cocaine-dependent subjects compared to controls, along with no difference between groups in the first eigenvalue, may indicate impaired myelin [26], [27], [28], [54], although this interpretation may not always be valid [29]. This is consistent with the finding of a forty-percent reduction in myelin basic protein in the corpus callosum of rats exposed chronically to cocaine [69]. For the cocaine-dependent group in the present study, a reduction in the normal restrictions to diffusion across the axon (as putatively indicated by higher mean of the second and third eigenvalues) may contribute to reduced anisotropy, thus resulting in the lower FA values found in the cocaine group compared to control subjects.

Several unresolved issues remain for future studies to address. Foremost, it is unclear if these biological and decision-making deficits represent premorbid conditions, the sequelae of chronic cocaine use, or perhaps even polysubstance abuse, as most cocaine-dependent subjects had also abused at least one other drug during their lifetime. Longitudinal and non-human animal studies will be needed to clarify this difficult issue. In support of the potentially deleterious action of cocaine on white matter, using a rat model of chronic (28-day) cocaine administration, Narayana et al. [69] recently showed lower FA in cocaine-treated compared to saline-treated rats in the posterior corpus callosum along with a corresponding reduction in myelin basic protein. In addition, early post-natal cocaine exposure in rats showed altered volume (macrostructure) as a result of the accumulation of microstructural demyelination, evidenced by disruption of gender-specific patterns of development in corpus callosum [70].

Additional analyses were carried out to determine the possible role of confounding factors, including lifetime alcohol use, smoking status, and a 2.31 year difference between groups in mean education level. The implication of these supplemental analyses is that relationships among the DTI outcomes and the potential between-group confounders were probably due only to a clustering effect owing to demographic differences in education and alcohol use between the two groups; whereas group status (rather than alcohol use or education) predicted DTI outcomes. Although there was no difference in the DTI results between tobacco users and non-users, a limitation in this study is that this study did not have quantitative data regarding amount of tobacco use. Consumption of tobacco and alcohol are often correlated, and this relationship is particularly strong at high levels of use in which heavy drinkers are likely to be heavy smokers and vice versa [71]. Therefore, future studies in which subjects are matched on these variables, including education, lifetime alcohol use, and quantification of tobacco use, would be necessary to eliminate the confounding effects of these variables on the DTI results.

The interpretation of lower FA in the corona radiata in cocaine users compared to normal controls may be confounded by the possibility that within-voxel crossing fibers of different orientations in this region may cause artifactually low measured anisotropy [52], [58]. From a therapeutic perspective, a critical question is whether or not these biological and behavioral deficits are reversible. Determining if abstinence from drug use results in improvements in DTI parameters or improved decision making should be of central importance to addiction medicine. Perhaps most scientifically intriguing is the extent to which changes in these two domains co-vary following abstinence.

## Supporting Information

Table S1 Mean net score ±SEM across each of five 20-trial blocks and total net score on the Iowa Gambling Task for the cocaine-dependent and control groups.
(0.04 MB DOC)

Table S2 Pearson correlations among level of (a) education and (b) significant fractional anisotropy (FA) clusters, mean of the second and third eigenvalues (λ⊥) clusters, and Iowa Gambling Task (IGT) net score. Values in the second column represent the Pearson r score for all subjects, and (in parentheses) the r score within the cocaine and control groups, respectively. P-values are for all subjects, as there were no uncorrected significant p-values within each group. FDR = false discovery rate.
(0.04 MB DOC)
