# Supplementary material for: Diffusion Tensor Imaging and Decision Making in Cocaine Dependence
Source: PLoS One. 2010 Jul 16;5(7):e11591. doi: 10.1371/journal.pone.0011591 (PMC2905416; doi:10.1371/journal.pone.0011591)
Supplement: Table S1 — Mean net score ±SEM across each of five 20-trial blocks and total net score on the Iowa Gambling Task for the cocaine-dependent and control groups. (0.04 MB DOC) [file pone.0011591.s001.doc]

Table S1. Mean net score ±SEM across each of five 20-trial blocks and total net score on the Iowa Gambling Task for the cocaine-dependent and control groups.

| Group | Block 1 | Block 2 | Block 3 | Block 4 | Block 5 | Total |
| --- | --- | --- | --- | --- | --- | --- |
| Cocaine | -2.13 | 1.33 | -0.13 | -1.07 | -0.67 | -2.67 |
|  | ±2.52 | ±1.99 | ±2.57 | ±2.86 | ±1.80 | ±6.89 |
| Control | -3.72 | 5.22 | 3.33 | 5.33 | 5.56 | 15.39 |
|  | ±1.46 | ±2.10 | ±1.46 | ±1.95 | ±1.56 | ±5.61 |
