# Supplementary material for: Diffusion Tensor Imaging and Decision Making in Cocaine Dependence
Source: PLoS One. 2010 Jul 16;5(7):e11591. doi: 10.1371/journal.pone.0011591 (PMC2905416; doi:10.1371/journal.pone.0011591)
Supplement: Table S2 — Pearson correlations among level of (a) education and (b) significant fractional anisotropy (FA) clusters, mean of the second and third eigenvalues (λ⊥) clusters, and Iowa Gambling Task (IGT) net score. Values in the second column represent the Pearson r score for all subjects, and (in parentheses) the r score within the cocaine and control groups, respectively. P-values are for all subjects, as there were no uncorrected significant p-values within each group. FDR = false discovery rate. (0.04 MB DOC) [file pone.0011591.s002.doc]

Table S2. Pearson correlations among level of (a) education and (b) significant fractional anisotropy (FA) clusters, mean of the second and third eigenvalues (λ) clusters, and Iowa Gambling Task (IGT) net score. Values in the second column represent the Pearson r score for all subjects; and (in parentheses) the r score within the cocaine and control groups, respectively. P-values are for all subjects, as there were no uncorrected significant p-values within each group. FDR = false discovery rate.

| Variable | Pearson r | Uncorrected p value | FDR-corrected p value |
| --- | --- | --- | --- |
| FA cluster 1 | .402 (-.054, .273) | .020 | .076 |
| FA cluster 2 | .354 (.206, -.107) | .043 | .076 |
| λ⊥ cluster 1 | -.383 (-.008, -.118) | .028 | .076 |
| λ⊥ cluster 2 | -.225 (.175, .188) | .207 | .207 |
| λ⊥ cluster 3 | -.369 (-.273, .067) | .036 | .076 |
| λ⊥ cluster 4 | -.315 (.024, .110) | .074 | .103 |
| IGT net score | .256 (.264, -.105) | .151 | .176 |
